# Neurodegenerative Disorders in Criminal Offending and Cognitive Decline Among Aging Inmates

**DOI:** 10.3390/neurosci6010005

**Published:** 2025-01-13

**Authors:** Sara Veggi, Fausto Roveta

**Affiliations:** 1 Department of Psychology, University of Turin, 10124 Turin, Italy; sara.veggi@unito.it; 2 Department of Neuroscience “Rita Levi Montalcini”, University of Turin, 10126 Turin, Italy

**Keywords:** Alzheimer’s disease, frontotemporal dementia, cognitive impairment, dementia, prison, offenders

## Abstract

Dementia, including Alzheimer’s disease (AD) and frontotemporal dementia (FTD), presents critical challenges for correctional systems, particularly as global populations age. AD, affecting 60–80% of dementia cases, primarily impairs memory and cognition in individuals over 65. In contrast, FTD, rarer than AD but not uncommon in those under 65, affects the frontal and temporal brain regions, leading to deficits in social behavior, language, and impulse control, often resulting in antisocial actions and legal consequences. Behavioral variant FTD is especially associated with socially inappropriate and impulsive behaviors due to frontal lobe degeneration. The prevalence of cognitive impairment in incarcerated populations is high, exacerbated by prison environments that compound distress and limited access to specialized healthcare. Studies indicate that up to 11% of United States state prison inmates over the age of 55 exhibit cognitive impairments, often undiagnosed, resulting in punitive rather than rehabilitative responses to symptoms like disinhibition and aggression. Ethical concerns around criminal responsibility for individuals with dementia are increasingly prominent, particularly regarding their ability to comprehend and engage in legal proceedings. The growing elderly prison population necessitates reform in correctional healthcare to include early cognitive assessment, targeted intervention, and tailored post-release programs. Addressing these needs is essential to ensure appropriate treatments, alleviate healthcare demands, and support reintegration for cognitively impaired inmates.

## 1. Introduction

Dementia, characterized by a progressive decline in multiple cognitive domains that disrupts daily social and occupational functioning, has become a significant and growing global health concern [1]. Currently, an estimated 6.5 million people in the United States are living with Alzheimer’s disease (AD), the leading cause of dementia [2]. Globally, approximately 47 million people are affected by dementia, with projections suggesting that this number may increase to 131 million by 2050, driven by rising life expectancy and aging demographics [3].

Together, AD and frontotemporal dementia (FTD) impose a considerable burden, affecting quality of life for individuals and families and presenting increasing demands on healthcare and social systems, contributing substantially to social and economic challenges in Western societies [4,5].

FTD, the most common presenile dementia syndrome after AD, comprises a spectrum of neurodegenerative disorders that lead to progressive deterioration of the frontal and temporal brain regions [6,7]. Both FTD and AD arise from a combination of genetic predisposition and environmental factors, which trigger the accumulation of abnormal proteins central to disease pathogenesis. Environmental stressors, including head trauma, cardiovascular risk factors, and lifestyle choices, further exacerbate disease progression [8,9,10].

The proteinopathies involved in FTD primarily include tau or TDP-43 pathology, while AD is characterized by amyloid-β plaques and neurofibrillary tau tangles. The accumulation of these misfolded proteins disrupts normal cellular function, leading to mitochondrial dysfunction, oxidative stress, and neuroinflammation [11,12,13]. These processes progressively damage neurons and their supporting glial cells, resulting in atrophy of critical brain regions.

In FTD, the pathology varies but consistently results in deficits aligned with damage to frontal and temporal brain areas, causing behavioral, linguistic, and executive function impairments [14,15,16].

While FTD represents only 5–15% of all dementia cases, it is prevalent among individuals under 65, making it a significant contributor to early-onset dementia [17]. Conversely, AD, the most common form of dementia, primarily affects individuals over 65 and accounts for 60–80% of all dementia cases [18].

The progression of neuronal damage in AD occurs over years, with the hippocampus and temporoparietal cortex being particularly vulnerable. This results in impairments in episodic memory, spatial orientation, and executive functioning. In contrast, degeneration of the frontal and anterior temporal lobes in FTD disrupts the neural networks responsible for behavioral regulation, emotional control, and social cognition [19,20]. Unlike FTD, which often first affects behavior and language, AD typically develops with mild memory impairment and gradually advances to severe cognitive and functional deficits [21].

Among FTD subtypes, behavioral variant frontotemporal dementia (bvFTD) is particularly impactful due to its effect on social and moral cognition, caused by frontal lobe degeneration [22,23,24]. The loss of neurons in the orbitofrontal cortex, anterior cingulate cortex, and related fronto-limbic pathways leads to disinhibition, impulsivity, apathy, and emotional blunting. For example, inappropriate behaviors, such as sexual advances, compulsive habits, or indecent exposure, arise because of impaired inhibitory control. Similarly, damage to anterior temporal regions reduces empathy and emotional insight, worsening the individual’s ability to interpret social cues [25,26,27].

bvFTD is frequently characterized by significant changes in personality, mood, and behavior, often appearing as the earliest noticeable symptoms before cognitive decline [28]. In certain cases, criminal behavior, particularly in individuals over 50, may be the first indicator of the disease [29]. Importantly, 14% of bvFTD patients were initially referred for diagnosis due to criminal conduct [30], with rare but severe offenses, such as homicide, also documented in the literature [31].

These neurodegenerative disorders pose profound challenges within the aging prison population—a demographic issue particularly impacting countries such as the United States, the United Kingdom, and Australia [32]. Research indicates that dementia prevalence rates reach up to 11% among older inmates in United States state prisons, with many diagnoses occurring post-incarceration due to inadequate screening and limited awareness among correctional staff [33]. Additionally, Kuffel et al. [34] noted that many elderly inmates exhibit mild cognitive impairment (MCI) even before incarceration, suggesting that early intervention could reduce recidivism among cognitively vulnerable individuals.

In this review we provide an overview of the current evidence on cognitive impairments affecting both the likelihood of offending and the aging process during detention, with a particular focus on neurodegenerative disorders such as AD and FTD.

## 2. Antisocial Behavior in Neurodegenerative Dementias

A significant association exists between certain dementia forms and antisocial behavior, particularly in bvFTD [35]. The degeneration of frontotemporal brain regions, crucial for impulse control and social behavior, often results in socially inappropriate conduct in bvFTD, such as theft or assault, underscoring how compromised impulse regulation and social judgment increase the risk of offending [36,37,38].

Frontal lobe dysfunction plays a critical role in linking dementia with antisocial behaviors [39,40,41]. Liljegren et al. [42] observed significantly higher rates of criminal behavior (42% vs. 14.9%) and socially inappropriate conduct (74.8% vs. 56.4%) in patients with FTD compared to those with AD, with criminal acts recurring more frequently in FTD (82%) than AD (53.3%). Non-tau pathologies, such as TDP-43, were nine times more associated with criminal behavior in FTD than tau pathology.

Criminal behavior in AD tends to emerge predominantly in the middle stages of the disease, when memory deficits, confusion, and disorientation begin to affect judgment and lead to non-violent offenses, such as trespassing, financial mismanagement, or traffic violations [43]. In the later stages, the cognitive decline is so severe that individuals often lose the capacity to engage in any form of coordinated criminal behavior, with behaviors being restricted to confusion-driven acts [44].

FTD presents a more multifaceted scenario. In the early stages, where frontal lobe degeneration is still developing, patients often retain physical and cognitive abilities but exhibit significant disinhibition, leading to impulsive and socially inappropriate behaviors, including theft or public misconduct [45]. As the disease progresses and frontal lobe damage becomes more extensive, impulsivity and aggression may lead to more severe antisocial actions, such as physical assault or property crimes. However, in advanced stages, similar to AD, severe executive dysfunction and physical debilitation limit the capacity for complex or violent criminal acts, with behaviors often restricted to outbursts or isolated aggressive incidents [46].

Previous work by Liljegren et al. [47] similarly found criminal behavior to be more prevalent in patients with bvFTD and semantic variant primary progressive aphasia (svPPA) than in AD. Specifically, criminal behaviors appeared in 37.4% of bvFTD patients, 27.0% of svPPA patients, and only 7.7% of AD patients. These behaviors varied by diagnosis. bvFTD patients engaged in a range of criminal acts, including financial mismanagement, traffic violations, unsolicited sexual advances, stalking, trespassing, and public urination. In svPPA, criminal conduct was largely restricted to theft, often rooted in compulsive tendencies or attraction to objects. The stages at which these behaviors manifest align with disease progression, as early and mid-stage FTD retains a higher risk of impulsive actions, whereas later stages show diminished capability for behaviors requiring organization or deliberate effort, due to profound cognitive decline.

Frontal lobe damage, which is prominent in bvFTD, is closely linked to increased impulsivity and aggression, manifesting as socially transgressive actions like shouting, name-calling, hitting, pushing, and biting [48]. This neurobiological underpinning for antisocial behavior in dementia is further evidenced by findings that approximately 54% of individuals with bvFTD have committed criminal offenses [49]. Subsequent research, including recent findings by Kumfor et al. [50], reinforces that FTD—especially bvFTD and right temporal variant frontotemporal dementia (rtvFTD)—disproportionately correlates with antisocial behaviors, including physical assault, financial recklessness, and inappropriate interactions in personal relationships. Notably, nearly half of dementia patients display such behaviors, with 19.1% officially encountering law enforcement. These patterns are likely driven by frontotemporal network deterioration, which impairs social judgment. Findings from Rainero et al. [51] and Scarpazza [52] support this by identifying frontal lobe degeneration as a key factor in the onset of acquired antisocial tendencies, which often appear abruptly in individuals with no prior history of such behaviors. This contrasts with other neurocognitive disorders like AD, where antisocial actions may stem more from confusion or memory issues than impulsivity or disinhibition. Such distinctions underscore unique behavioral patterns across dementia subtypes, with FTD-linked behaviors frequently resulting in legal repercussions due to compromised impulse control and executive function [53,54].

Recent findings by Ginters et al. [55] reinforce this association, showing that although individuals with neurocognitive disorders generally commit fewer crimes than the broader population, those with FTD—particularly males—demonstrate a significantly higher proclivity toward criminal behavior compared to AD patients. In their study, offenses were committed post-diagnosis by 7.2% of men and 2.0% of women with FTD, compared to 2.8% of men and 0.4% of women with AD. Traffic violations were the most prevalent offenses across both groups, but FTD patients showed significantly higher rates of property and violent crimes, likely due to severe executive dysfunction and disinhibition associated with frontal lobe degeneration.

These neurodegenerative changes in FTD and AD highlight the critical role of impaired self-regulation and moral reasoning in driving offending behaviors, even when individuals maintain sufficient awareness of their actions and recognize moral and social norms [56]. In AD, memory and orientation deficits often lead patients to non-violent offenses, such as theft. Conversely, vascular dementia is more frequently associated with violent acts, including assault and arson. FTD, which involves frontal lobe damage, is strongly linked to impulsive behaviors like aggression and socially inappropriate attentions, underscoring how cognitive impairments drive antisocial actions differently across dementia subtypes [30,57,58]. In FTD and other frontal-lobe dementias, symptoms like disinhibition and dysexecutive syndrome play a significant role in fostering impulsive, unplanned behaviors [59]. Supporting this, Ginters et al. [55] found impulsive offenses more common among FTD patients, particularly males. Despite these tendencies, crime rates remain lower in neurocognitive disorder populations than in the general population, as mortality and cognitive decline limit the capacity to engage in elaborated criminal behaviors.

Furthermore, alcohol-related cognitive impairments, such as Wernicke–Korsakoff syndrome (WKS), amplify the propensity for antisocial behavior, with offenses often involving aggression or vandalism. Offending rates in individuals with WKS are generally higher prior to diagnosis and tend to decrease afterward [57]. The interplay of substance abuse and cognitive impairment increases the risk of impulsive criminal behaviors, adding nuances to the link between neurocognitive disorders and antisocial tendencies [60].

## 3. Prevalence of Cognitive Impairment Among Incarcerated Individuals

Cognitive impairment rates among prison populations are disproportionately high, far exceeding those in the general community [61]. The aging prison population intensifies the demand for healthcare, as older inmates often suffer from chronic conditions requiring more frequent and specialized medical care. However, healthcare delivery in correctional settings has struggled to keep pace with these demographic changes, contributing to more acute health events and higher costs associated with treating elderly inmates outside prison facilities [62].

The impact of cognitive impairment within this aging group is significant. Ahalt et al. [63] found that 70% of incarcerated individuals over age 55 scored below 25 on the Montreal Cognitive Assessment (MoCA), indicating the presence of MCI. This impairment not only heightens the risk of hospitalization but also correlates with higher recidivism rates and difficulties in adhering to legal procedures, thus underscoring the urgent need for targeted interventions within correctional settings.

As the elderly inmate population grows, these issues are aggravated by resource gaps in mental healthcare provision. Older inmates, already fragile due to cognitive and physical issues, often receive suboptimal medical care, leading to poorer health outcomes compared to non-incarcerated individuals [64]. Similarly, substandard care contributes to higher mortality rates and exacerbates critical risk situations associated with criminal behavior upon release (e.g., interpersonal isolation, lack of financial support, inappropriate housing) [65,66].

Studies across various countries support these findings, highlighting the high prevalence of cognitive disorders among older inmates. For example, research in Sweden shows that older individuals with criminal backgrounds face a significantly higher risk of dementia and MCI than the general population [61]. Similar trends are seen in the United Kingdom, where up to 25% of inmates over age 55 have undiagnosed dementia [67]. Cox and Wallace [68] also report that formerly incarcerated individuals are 2.7 times more likely to develop dementia, attributed to factors such as chronic stress, low educational attainment, and pre-existing cognitive deficits, which are disproportionately common in prison populations.

### Risk Factors for Cognitive Decline in Prison Populations

The high rates of cognitive decline among aging inmates can be attributed to an intricate interplay of biological and environmental factors that are often triggered within prison environments. Aging is commonly linked to a gradual decline in cognitive function; however, for incarcerated individuals, this decline seems to progress more rapidly, with their physiological age estimated to be 10–15 years beyond their chronological age [69].

This accelerated aging process in prisons is closely associated with common lifestyle and environmental factors within correctional facilities. For instance, the high prevalence of smoking, physical inactivity, and poor nutrition among inmates is well-known [70]. Additionally, the chronic stress associated with incarceration—from uncertainty, stigma, and lack of autonomy—further affects vascular health, contributing to early cognitive impairment typically observed in much older individuals within the general population [71,72].

Environmental stressors specific to prison settings also have a significant impact. Overcrowded facilities, frequent exposure to violence, and the pervasive sense of loneliness add layers of psychological and physiological stress [73,74,75], each amplifying the effects of aging. These conditions have been shown to precipitate earlier onset of age-related diseases, including cognitive decline, when compared to the general population, who may not face these cumulative stress factors [32,76]. In this sense, such early onset of cognitive impairment is not merely a result of biological aging but is a reflection of the harsh situational factors within the penitentiary system that, over time, become part of the inmates’ lived experience.

Adding to this burden, traumatic brain injuries (TBIs) are significantly more common among the prison population, affecting 25% to 87% of incarcerated individuals compared to approximately 8% of the general population [77]. TBIs are closely related to a heightened likelihood of cognitive impairment and dementia, highlighting another risk factor specific to correctional environments [71]. Substance abuse, which is prevalent within many prison populations, further worsens the risk of cognitive decline. Research has shown that chronic substance abuse is correlated with long-term cognitive impairments, including early-onset dementia, due to the neurotoxic effects of drugs and alcohol [78,79].

The psychological strain of incarceration also has far-reaching implications for cognitive health. Incarcerated individuals often experience stronger emotional stress, stemming from the isolation from family, restricted personal freedom, and limited control over their daily routines [80,81,82]. This prolonged stress has been shown to negatively affect cognitive performance and overall mental health, with impacts ranging from short-term memory impairment to more significant, long-lasting cognitive deficits [83]. Furthermore, for inmates with pre-existing health conditions, these risks are increased by the limited access to rehabilitative services and mental stimulation within the correctional setting. The lack of opportunities for meaningful engagement and cognitive exercises can lead to accelerated decline, as regular cognitive stimulation is essential for maintaining brain health [84,85].

## 4. Aging and Cognitive Decline During Detention

In the United States, the proportion of state prisoners aged 55 and older increased dramatically from 3% to 10% between 1993 and 2013—a rise of over 400%—primarily driven by policies associated with mass incarceration and “tough on crime” initiatives [62]. These strategies emphasize longer custody, delayed parole, and strict sentencing policies, such as “three strikes” laws, with limited alternatives to imprisonment [86,87]. As a result, these measures have significantly altered prison demographics, disproportionately affecting older individuals and contributing to a “prison boom” in a criminal justice system that often prioritizes punitive measures over rehabilitative approaches [88,89,90]. By 2030, a substantial proportion of United States inmates are projected to be over 55—a trend also seen in Italy and the United Kingdom, where the inmate population over 50 has tripled in the past two decades [67,79]. This aging demographic faces complex health issues, including rising rates of cognitive decline, such as MCI and dementia [91].

The high-stress prison environment, along with limited access to specialized healthcare, often heightens cognitive issues among aging inmates, while correctional facilities remain under-resourced to meet the growing needs of those with dementia [92]. Also, dementia within prison populations is frequently underreported, with United States estimates ranging from 1% to 44% [93]. In this regard, only 15.4% of MoCA-diagnosed dementia patients had a dementia diagnosis documented in their medical records in a study by Baillargeon et al. [94], and this further complicates efforts to provide adequate care. Research has linked the impact of environmental deprivation in prisons to worsening cognitive deficits. For instance, Meijers et al. [95] found that prison environments exacerbate cognitive dysfunction, including impaired working memory and diminished attention capacity—both essential for daily prison functioning and successful post-release reintegration. Additionally, a history of incarceration independently raises the risk of cognitive impairment in later life, reaching a level comparable to the genetic risk associated with the APOE-ε4 allele; this risk is dose-responsive, with longer periods of incarceration linked to greater cognitive decline, highlighting incarceration as a significant risk factor for age-related neurodegeneration [96].

Studies show that older inmates experience a substantially higher burden of both mental and physical health conditions compared to both younger inmates and the general aging population. For example, among older adults with a history of incarceration, the prevalence of issues such as mobility limitations, urinary incontinence, sensory impairments, and chronic lung disease is 20% to 80% higher [54]. Furthermore, around 40% of older inmates report chronic conditions, including hypertension, diabetes, arthritis, and liver disease, as well as significant mental health issues like anxiety, depression, and a heightened fear of or desire for death [78,97]. 

A lack of structured protocols for dementia screening and management in many correctional systems compounds these difficulties [98]. For example, Combalbert et al. [99] documented that nearly 20% of older male inmates in France exhibit moderate to severe dementia, with an even larger proportion showing signs of cognitive impairment. Due to inadequate resources and the absence of healthcare services tailored to cognitive health, these issues often go unaddressed [100,101]. Research suggests that executive dysfunction in older inmates, characterized by deficits in self-regulation and impulse control, increases the likelihood of antisocial behaviors, which can lead to violent incidents (e.g., explosive rage) and further disrupt the prison climate [102].

Further exacerbating these cognitive health issues are prison-specific stressors, inadequate living conditions, and limited mental stimulation, all of which accelerate cognitive decline among incarcerated individuals [99]. Chronic stress, a suboptimal diet (e.g., food not suited to individual medical conditions), and restricted access to healthcare contribute to premature aging within prisons, increasing the risk of cognitive impairment and dementia [94,103]. Researchers argue that this healthcare gap represents a critical failure within the correctional system, advocating for integrated prison healthcare services that align more closely with broader public health systems to meet the unique needs of older, cognitively impaired inmates [97].

## 5. Barriers to Diagnosing and Managing Cognitive Impairment in Correctional Facilities: Resource Limitations, Diagnostic Challenges, and Post-Release Implications

The timely diagnosis of cognitive impairment within prison populations is significantly hindered by multifaceted obstacles, including substantial resource constraints, limited access to specialized healthcare, and an acute shortage of adequately trained personnel. In correctional settings, symptoms of cognitive impairment, such as memory loss, disorientation, and agitation, are frequently misinterpreted as non-compliance or disciplinary issues rather than medical conditions, resulting in their frequent oversight [104,105]. Consequently, inmates with cognitive impairments often receive punitive responses rather than necessary clinical interventions, a misalignment that accelerates their cognitive decline and may lead to detrimental impacts on their overall health and well-being [106,107]. This issue is further perpetuated by a lack of standardized protocols for dementia diagnosis and cognitive assessment within correctional facilities, leaving many inmates undiagnosed and, consequently, untreated [108].

According to Brooke et al. [93], many correctional institutions fail to incorporate dementia-specific screening tools, resulting in high rates of underdiagnosis and mismanagement of inmates with cognitive impairments. Only 30% of prisons in England and Wales, for example, have established protocols for routine cognitive evaluations [107], and correctional facilities often lack access to trained geriatric and neurology specialists [66], with about 96% of United States state facilities not offering dedicated geriatric healthcare [84]. These findings reveal a systemic neglect in the approach to inmate cognitive health.

While diagnostic tools such as the MoCA are commonly employed in correctional settings, they may lack the adaptability to address the psychological and environmental factors prevalent within prison environments [106]. Such limitations underline the critical need for developing and validating cognitive screening tools that are specifically adapted for incarcerated populations. Addressing these barriers through early detection and intervention could alleviate both the healthcare and legal burdens associated with cognitive impairment within these settings [61,67]. However, despite the clear benefits, many correctional systems lack the requisite infrastructure to implement comprehensive cognitive health assessments. This results in a continual decline in the health of cognitively impaired inmates, who often face severe deterioration due to a lack of timely intervention [109].

Optimal care for older prisoners with dementia requires a multifaceted approach that includes regular cognitive assessments for early detection, specialized medical and housing services to address demanding needs, and structured activities to support both cognitive and physical health.

Beyond assessment and diagnosis, correctional facilities struggle with the ongoing task of managing inmates with cognitive impairments. Christodoulou [71] proposes that prisons could play a role in addressing modifiable dementia risk factors, such as through dietary improvements and physical activity programs; however, implementing these interventions is limited by resource constraints, security concerns, and logistical barriers. Multidisciplinary care pathways for dementia—encompassing early detection, comprehensive treatment, and sustained support—present a promising yet underutilized strategy in correctional settings [67,101]. In the absence of such specialized care, prisons risk evolving into de facto long-term care facilities for aging inmates with cognitive decline, with inefficiencies leading to costs averaging USD 70,000 per inmate annually—two to three times that for younger inmates [32,63,110]. Meanwhile, adapting prison facilities with accessibility modifications, such as grab bars, shower chairs, and age-segregated housing units, could alleviate immediate pressure, especially when paired with early release options for low-risk elderly inmates [110,111]. Furthermore, comprehensive geriatric and palliative care training for correctional staff and younger inmates remains essential to support functional limitations and foster a safer, more accommodating environment for this vulnerable population.

The health implications for inmates with cognitive impairments extend well beyond incarceration, as many individuals encounter substantial barriers to reintegration into society after serving their sentences. Upon release, these former inmates often experience difficulties with essential daily tasks, such as managing finances and maintaining social relationships, which can impede their successful reentry [26,86]. In the absence of structured community support, they are at increased risk of adverse outcomes, including homelessness and recidivism [101]. While some community-based programs offer housing and healthcare services tailored to the needs of former inmates with cognitive impairments, these initiatives remain sparse and geographically limited [67]. This scarcity underscores an urgent need for the development and expansion of comprehensive reentry programs specifically designed to address the unique needs of individuals with cognitive impairments. Establishing such programs could serve as a critical intervention point, bridging the gap between correctional and community care and fostering improved outcomes for this vulnerable population (Table 1).

## 6. Ethical and Legal Considerations

Ethical concerns regarding the criminal responsibility of individuals with dementia become especially pronounced in the later stages of the disease, where cognitive impairments significantly hinder comprehension and self-control. Hallich [112] argues that punishing individuals with late-stage dementia raises profound moral dilemmas, as they often lack the capacity to understand their actions or the rationale for their punishment. Studies by Anderson and Baird [113] and Chaguendo-Quintero et al. [114] further highlight that sentencing elderly inmates with dementia introduces complex legal and ethical issues about accountability, questioning the fairness of prolonged sentences when cognitive impairments limit personal responsibility. In line with this, Reutens et al. [115] found that 67.7% of offenders with dementia were deemed unfit to stand trial or were acquitted due to cognitive impairment, with many cases citing dementia as a mitigating factor, resulting in reduced sentences. As dementia progresses, inmates’ ability to engage meaningfully in legal proceedings deteriorates, raising serious concerns about their competence, agency, and the fairness of continued incarceration [76]. These issues are exacerbated by evidence indicating that many older inmates, owing to typical age-related declines in criminal behavior, generally pose a significantly lower overall risk of reoffending [116,117]. Nevertheless, early release policies, such as compassionate parole, remain underutilized. Psick et al. [62] advocate for the expansion of these measures, arguing that they could better address the needs of aging inmates with cognitive impairments. However, restrictive eligibility criteria and bureaucratic delays often prevent the timely release of terminally ill or severely impaired prisoners. Fazel et al. [98] emphasize the ethical complexities of detaining individuals who develop dementia while incarcerated, highlighting that the legal system frequently fails to consider their diminished cognitive capacities, raising questions about the unnecessary criminalization of dementia-related behaviors. Additionally, Hallich [112] stresses that punishing those unable to comprehend their crimes conflicts with both retributive and deterrence-based justice models. These ethical concerns underscore the need to reassess sentencing and incarceration policies for cognitively impaired inmates.

The intersection of cognitive impairment and the criminal justice system is particularly troubling for racially marginalized groups, who disproportionately face health disparities. Structural inequities in healthcare and education not only foster cognitive decline but also limit individuals’ ability to understand legal processes, further exacerbating their frailty [68]. As dementia progresses, inmates may lack the mental capacity (mens rea) required for criminal culpability. Arias and Flicker [118] explain that dementia impairs essential cognitive functions—such as memory, judgment, and decision-making—often leading to behaviors that appear intentional but are unintentional in nature. These impairments reduce an individual’s understanding of their actions and the associated consequences, an essential element of criminal responsibility.

Kapp [119] argues that dementia-related behaviors should be understood through a public health lens rather than treated as criminal acts. This perspective shift is especially relevant as more cognitively impaired individuals reside in community-based settings due to evolving long-term care policies, which increases their risk of criminal justice involvement. Such a shift also addresses family concerns about liability when a dementia patient causes harm, as in cases involving access to a family member’s firearm, balancing the need for accountability with supportive care.

The ethical dilemma of incarcerating individuals who can no longer grasp the reasons for their punishment points to the urgent need for alternative legal frameworks. Arias and Flicker [118] propose that categorical protections similar to those afforded to juveniles and individuals with psychiatric disorders should be extended to inmates with dementia, ensuring that their diminished capacity is acknowledged in criminal proceedings. Kapp [119] supports this perspective, advocating for a more substantial role of elder law attorneys in cases involving cognitively impaired defendants, given their expertise in the complexities of aging. Additionally, he emphasizes that prosecutors, who wield significant discretion, should be better informed about dementia’s effects to divert such cases away from criminal prosecution and toward public health interventions [109]. Cipriani et al. [84] similarly question the suitability of traditional incarceration for individuals with severe cognitive decline, advocating for alternative sentencing options such as medical parole or placement in specialized care facilities, which would more ethically meet the needs of inmates with advanced dementia. Adding to these concerns, Arias and Flicker [118] highlight the human rights issues associated with incarcerating those who can no longer comprehend their legal circumstances. Indeed, the justice system has a moral and legal obligation to adapt its policies to prevent the undue punishment of cognitively impaired individuals. Diversion programs focusing on treatment rather than imprisonment, particularly for cases where dementia-related behaviors result in unintentional law-breaking, more closely align with principles of justice and human dignity, thereby reducing the risk of wrongful incarceration [118].

In this context, Kapp [119] highlights the importance of addressing the financial and social frameworks in which individuals with dementia live and receive care, by emphasizing that any realistic public health-oriented alternatives to incarceration should take into account the existing infrastructure for long-term care funding (including private insurance and family contributions). Recognizing these financial factors is paramount for developing sustainable alternatives to incarceration for this population.

## 7. Conclusions

Criminal behavior among individuals with neurocognitive disorders, such as dementia and FTD, presents a growing and complex issue, especially as the global population ages. Cognitive decline in these individuals often leads to impaired judgment, reduced impulse control, and compromised executive function, contributing to socially inappropriate or even criminal actions. In fact, prefrontal cortex impairments, commonly associated with antisocial tendencies, are strongly linked to diminished moral judgment and impulse control [120]. This may explain why individuals with neurocognitive disorders may engage in rule-breaking behaviors, often without intent to harm. As Talaslahti et al. [53] warn, emerging criminal behavior in older adults can be an early indicator of neurocognitive disorders, underscoring the need for medical evaluation when such behaviors arise.

A deeper understanding of these behaviors is essential for managing justice-involved individuals affected by neurodegenerative conditions effectively [55,59]. Equally important is recognizing the heterogeneity within this aging prison population, as these groups vary significantly in their criminal career and paths to incarceration. In fact, older incarcerated individuals include those who received lengthy sentences when young and have since aged within the prison system, recidivist offenders with a history of repeated offenses leading to multiple incarcerations, historical offenders convicted for crimes committed long before sentencing, and first-time offenders who committed their initial offense later in life [121]. Recognizing this variability is essential to developing correctional policies that address the unique healthcare needs of an aging prison population, including the urgent requirement for specialized dementia care pathways and targeted services. Without these tailored interventions, prisons are ill-equipped to meet the healthcare needs of an aging population with neurocognitive disorders [64,93].

Originally designed for young, able-bodied inmates, correctional facilities now risk becoming ineffective healthcare providers for individuals with dementia and other neurocognitive disorders due to inadequate governance strategies [32,101]. Therefore, it is crucial for penitentiary systems to prioritize comprehensive approaches for screening, diagnosing, and managing cognitive impairments within their aging populations to alleviate strain on these institutions.

Future research should aim to design individualized interventions and reform policies to ensure humane treatment and adequate care for this group. Implementing appropriate resources and strategies may enhance the quality of life for aging inmates while concurrently addressing the broader societal impacts of incarceration associated with cognitive decline [70,101].

## 8. Search Strategy

This manuscript is based on a non-systematic review and analysis of recent high-quality articles addressing cognitive impairments and neurodegenerative conditions within correctional systems. The main objective is to highlight the unique burden posed by these disorders, while discussing their implications for criminal responsibility and ethical considerations in prison settings. Relevant references were selected from PubMed, the Web of Science, or Scopus. Search terms included “Cognitive Impairment”, “Neurodegenerative Disorders”, “Prison Population”, and “Aging Inmates”. Additional articles were identified by reviewing the bibliographies of relevant English-language papers.

## Figures and Tables

**Table 1:** Overview of neurodegenerative disorders, symptoms, and care challenges in aging inmate populations.

Dimension	Description and Data	Main References
Prevalence of Neurodegenerative Disorders in Aging Inmate Populations	**Alzheimer’s Disease (AD)**: 60–80% of dementia cases globally, primarily affecting those over 65. **Frontotemporal Dementia (FTD):** 5–15% of all dementia cases, more common in individuals under 65. Behavioral variant FTD (bvFTD) is associated with socially inappropriate behaviors due to frontal lobe degeneration. **Mild Cognitive Impairment (MCI):** present in up to 11% of U.S. prison inmates over 55, with prevalence rates significantly higher than in non-incarcerated aging populations.	[1,17,63]
Symptoms and Behaviors Associated with Different Dementia Types	**Alzheimer’s Disease (AD)**: memory impairment, cognitive decline, confusion, leading to non-violent offenses, e.g., trespassing due to disorientation. **Behavioral variant FTD (bvFTD):** impulsivity, disinhibition, inappropriate social actions (e.g., theft, assault, unsolicited advances). **Vascular Dementia:** linked to higher aggression, with violent behaviors like assault often documented in prison settings.	[22,37,47]
Impact of Neurodegenerative Disorders on Inmate Behavior and Legal Implications	**Impulse Control and Disinhibition:** frontal lobe degeneration in FTD leads to impulsivity, aggression, and boundary-crossing behaviors. **Criminal Responsibility:** ethical questions around culpability, especially for bvFTD and AD patients exhibiting antisocial behaviors without intent. **Parole/Compassionate Release:** high re-offense risk without intervention, yet the cognitive impairment may justify release based on diminished capacity to participate in legal processes.	[32,35,76]
Challenges in Diagnosis and Care for Cognitively Impaired Inmates	**Diagnostic Limitations:** high rates of underdiagnosis due to lack of specialized screening tools in prison. MoCA (Montreal Cognitive Assessment) used but not tailored for correctional settings. **Healthcare Resource Constraints:** limited access to neurology and geriatrics in prisons, with undiagnosed dementia affecting up to 25% of inmates over 55. **Implications for Care:** insufficient infrastructure and trained personnel, leading to punitive responses to symptoms rather than clinical intervention.	[93,104,106]

## Data Availability

No dataset was generated or analyzed during the current study.
